# A grand mimicker of pulmonary malignancy: The massive mediastinal intercostal nerve tumour

**DOI:** 10.1002/rcr2.1422

**Published:** 2024-06-27

**Authors:** Syed H. Haq, Claire Friederick, David Eapen, Sidra Shah, Hoshimjon Begmatov, F. N. U. Sandesh, Sreenivasa Chanamolu

**Affiliations:** ^1^ Department of Internal Medicine BonSecour Mercy Health—St. Rita's Medical Center Lima Ohio USA; ^2^ Department of Pulmonology BonSecour Mercy Health—St. Rita's Medical Center Lima Ohio USA

**Keywords:** intercostal nerve, intrathoracic, neurogenic tumour, schwannoma

## Abstract

Mediastinal mass‐like manifestations often cause alarm and instigate a myriad of investigative testing to rule out insidious malignant processes. However, a unique and benign finding, the schwannoma can present either incidentally or while in pursuit of a symptomatic presentation. Given its rarity, limited literature exists on these neurogenic tumours with less than three dozen reported cases. No specific guidelines exist regarding the extent of required advanced imaging or degree of invasive evaluation. Therefore, practitioners confronted with these intrathoracic tumours may find management challenging or delayed. We present a case discussing a large benign tumour causing symptomatic burden, the investigative methods implored and treatment modality. We add to the literature another unique presentation of an intercostal nerve sheath tumour with schwannoma pathology.

## Graphical abstract

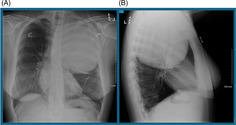

## INTRODUCTION

Schwannomas are nerve sheath tumours arising from the nerve bundles. These tumours can present anywhere in the body that have nerves coursing through, and therefore can erupt in a variety of locations. Commonly, they develop in the upper extremities and at the cerebellopontine angle intracranially.^1^ Pulmonary manifestations remain particularly rare, developing intrapulmonary or endobronchial.^2^ This stems from the fact that there is limited nerve distribution within the lungs. They especially show predominance in individuals 20–50 years of age and do not discriminate between genders.^3^ Furthermore, they are known to grow notoriously slow and relatively follow a benign course. However, given their insidious growth pattern and vast ability to develop at any location within the body, discovery is often delayed. As such clinical presentation can vary tremendously and at times increasing the complexity of management or even prolonging treatment. We will discuss such a case involving a peculiarly large symptomatic presentation of an intrathoracic intercostal nerve tumour with schwannoma pathology.

## CASE REPORT

**FIGURE 1:**
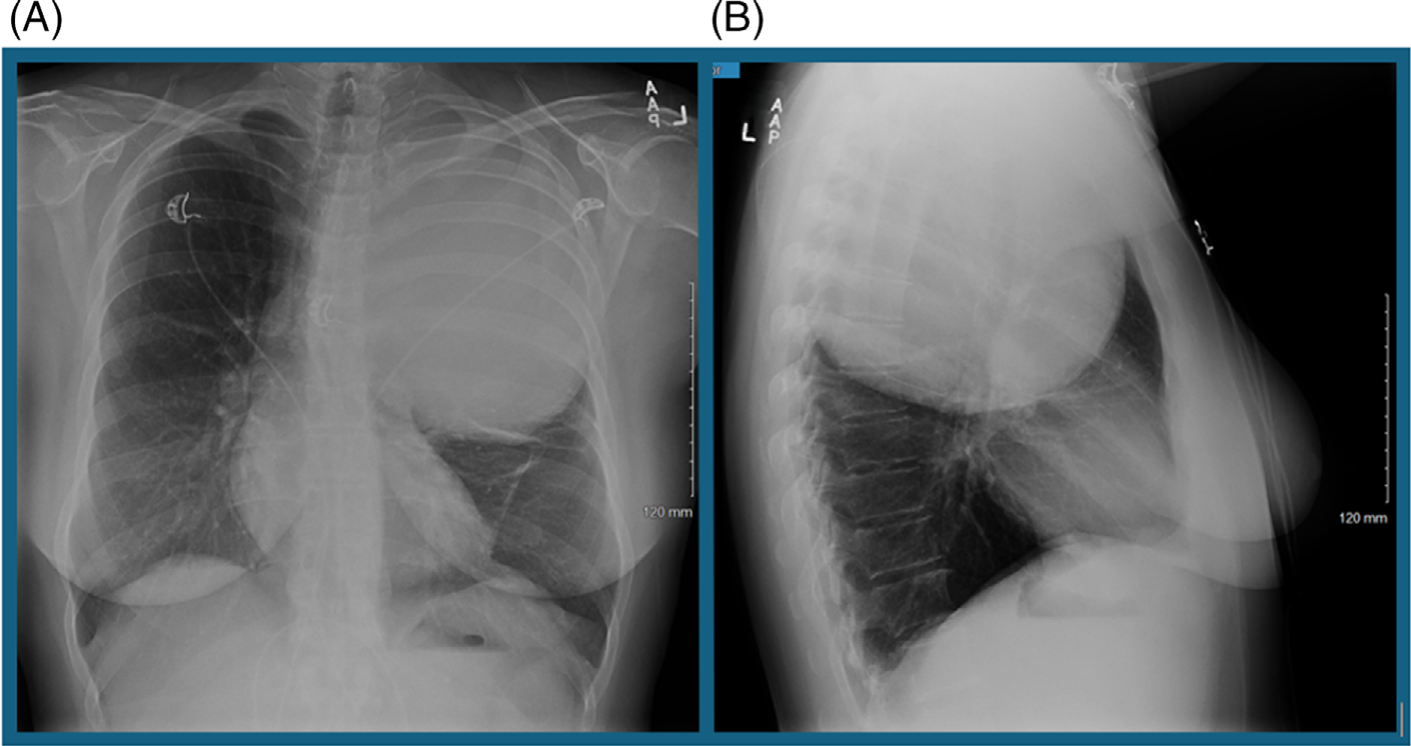
Chest x‐ray demonstrates 15 × 18 × 18 cm mass occupying the mid and upper left hemithorax with adjacent compressive atelectasis, rightward deviation of a normal calibre trachea.

**FIGURE 2:**
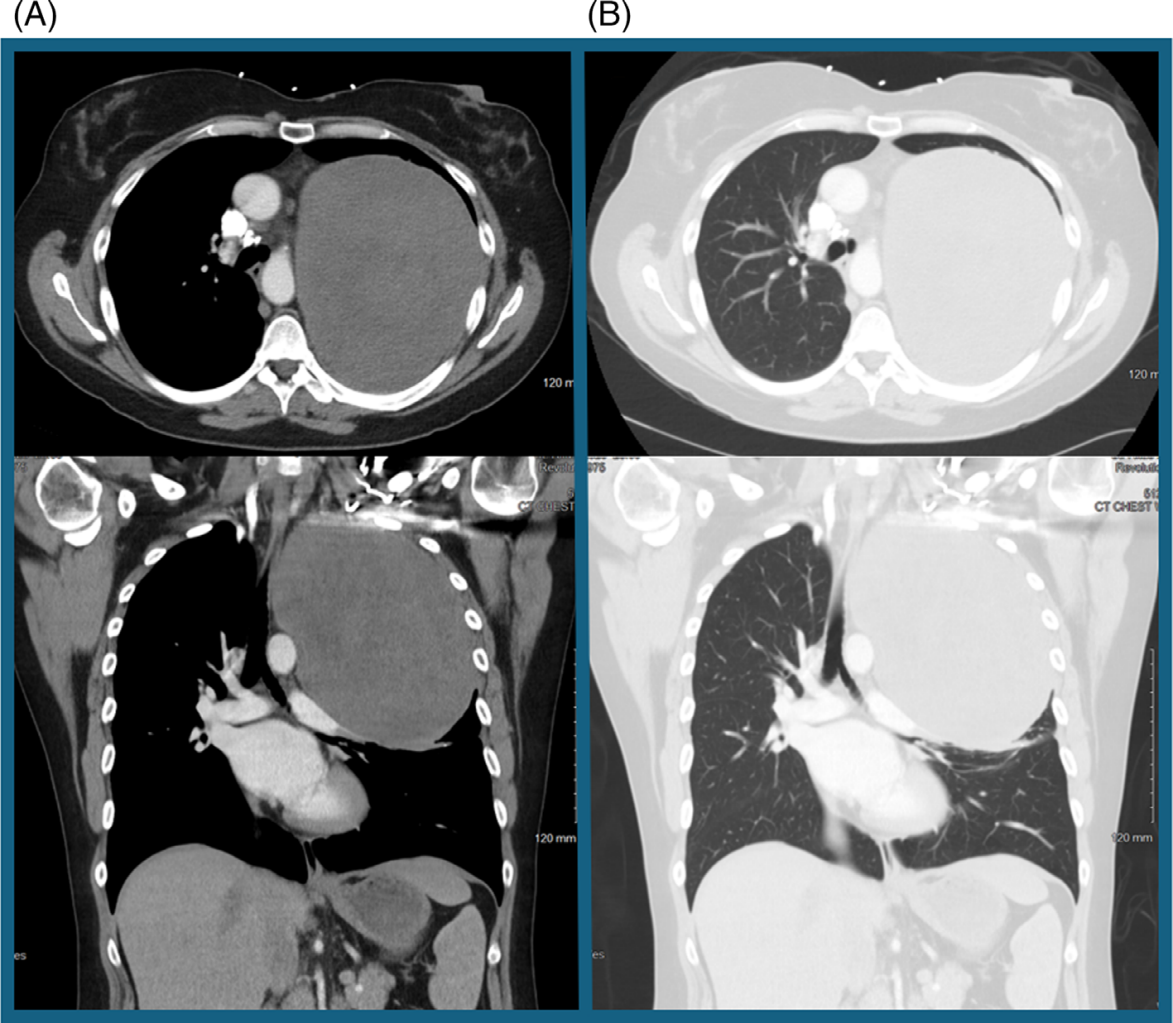
Computed tomography chest demonstrates a well encapsulated large left lung mass measuring 16 × 13 × 16 cm, without significant airway compromise, compressing the mediastinum and some extrinsic compression of the oesophagus.

A 42‐year‐old Caucasian female with a past medical history of hypertension, gastric reflux disease, migraines and anxiety presented to the emergency department for worsening intermittent dysphagia. She reported a sensation of food and liquids getting stuck, with associated chest discomfort but otherwise denied any overt pain. A chest x‐ray demonstrated a 15 × 18 × 18 cm mass occupying the mid and upper left hemithorax with adjacent compressive atelectasis, rightward deviation of a normal calibre trachea, as seen in Figure 1A,B. Given the left hemithorax mass, further investigation was sought with a computed tomographic (CT) chest that visualized a well encapsulated large left lung mass measuring 16 × 13 × 16 cm, without significant airway compromise, compressing the mediastinum and some extrinsic compression of the oesophagus, as seen in Figure 2A,B. Secondary to the mass size and location, the vascular supply was not clearly delineated on imaging. Initial considerations were for neoplasm, although no appreciable mediastinal lymphadenopathy was noted. The patient was inevitably admitted for further evaluation.

**FIGURE 3:**
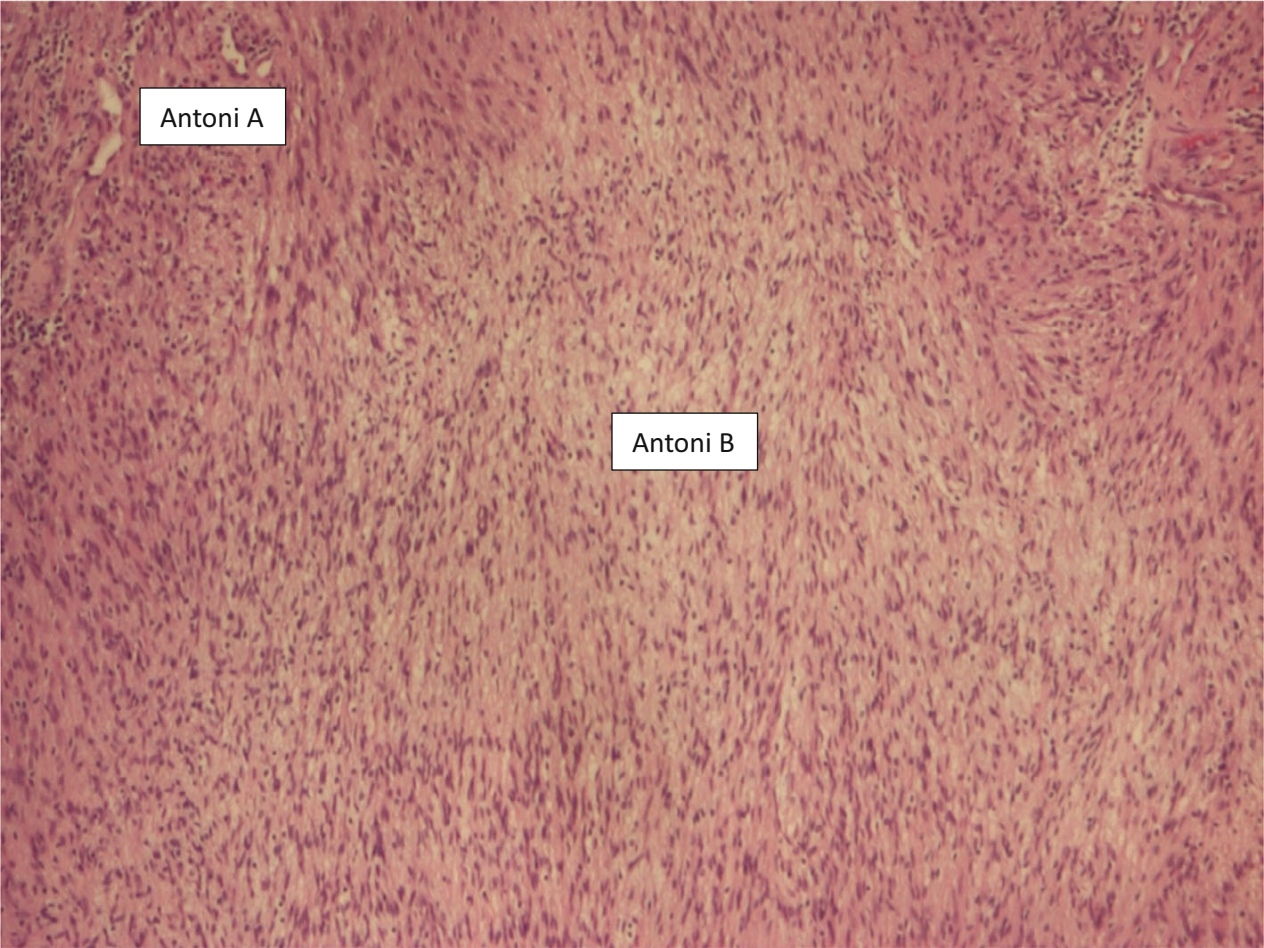
Intraoperative pathology showing evidence of schwannoma with both Antoni A and B pathology.

She underwent a CT guided biopsy with pathology consistent with cellular schwannoma. Although, the mass was deemed benign, she was considerably symptomatic with imaging elucidating mediastinal mass effect. Cardiothoracic surgery was consulted, and the patient was taken to surgery. Access was obtained with a posterolateral thoracotomy incision with the pleural space entered in the sixth intercostal space. Upon initial dissection, the mass was found to displacing 3/4th of the left pleural space with significant collapse of the left lung. It was adherent to the left upper lobe, the lateral and posterior chest wall, as well as the apex of the pleural cavity. The mass was noted to be supplied by the left subclavian artery. The left fifth rib was dissected and removed to facilitate access. The mass appeared intraoperatively to originate from the T2‐3 vertebral area from an intercostal nerve sheath. A difficult dissection was undertaken, complicated by damage to the subclavian artery at the apex as well as air leak requiring suture and an apical left upper lobe wedge resection. To facilitate improved respiration, a cryoablation of the left intercostal nerves for the 4th through 6th ribs was performed Upon removal, the left posterior mediastinal mass weighted 1.5 kg in its entirety. The patient was transferred to the ICU with two chest tubes in place. Post‐operatively, she underwent bronchoscopy with mucous plugging removed in the left upper lobe. She was extubated within 24 h of the procedure completion. Her chest tubes were removed after 2 weeks after a prolonged pulmonary rehabilitation regimen. Pathology again confirmed Schwannoma with positive S100 (Figure 3); additional adhesive tissue was deemed pulmonary in origin. A follow up MRI of the chest and T‐spine was negative for any intravertebral extension of this neurogenic tumour.

## DISCUSSION

As alluded to earlier, neurogenic tumours are relatively uncommon in the mediastinum, accounting for only 12%–21% of all mediastinal tumours. When they do arise, they are typically found in the posterior mediastinum for 90%–95% of cases.^4^ Particularly, schwannomas represent 25%–34% of neurogenic tumours.^3^ Most mediastinal schwannomas generally follow an asymptomatic course and as such, can go prolonged periods without detection. However, symptomatology may develop secondary to rapid growth or invasion of adjacent structures. These symptoms can be diverse, depending on the local structures affected, but often present as mimicking asthma due to airway obstruction. Regardless, once discovered it is imperative to definitively rule out a malignant process.

Imaging can provide good intuition regarding whether it is benign or malignant. Radiographically, they are spherical—but can be lobulated or dumbbell shaped, well encapsulated, and nearly always solitary. Usually, they appear homogenous but with larger schwannoma's heterogeneity may be seen because of haemorrhage or cystic degeneration. This is followed by CT or endobronchial ultrasound guided biopsy. Additionally, FDG‐PET can help differentiate between malignant and benign lesions, especially if concerning lymph nodes are present. With this modality schwannoma demonstrate low uptake.^5^ The American College of Radiology Appropriateness Criteria also indicates that magnetic resonance imaging with or without contrast may also be an appropriate tool for further evaluation of mediastinal masses. Histologically, two predominant patterns are seen. Antoni A is highly cellular with areas of spindle structured cells interweaved irregularly in a palisade‐like pattern, whereas Antoni B is a less cellular array of elongated cells composed in a loose myxoid distribution. They are shaped fusiform, eccentrically situated near the associated nerve, and enclosed in a capsule. These findings can appear nonspecific, providing a large differential, therefore immunohistochemical staining is beneficial. These markers include S‐100, Calretinin, Desmin, Vimentin, EMA, SMA, CD34, CD56 and CD 117.^2^

Schwannomas carry a relatively good prognosis, with rare probability for malignant transformation and requiring intervention only if symptomatic. Most neurogenic tumours are benign with 93.5% and only 6.5% progress to malignant variant.^3^ However, consideration for malignant transformation should be given depending on larger mass size, and presence of malignant features including greater cellularity and mitotic figures, local invasion, or bone destruction. Particularly, higher density of Ki67 expression correlated with in malignant peripheral nerve sheath tumour when compared to benign sheath tumours.^2^

Therefore, the mainstay treatment for schwannoma includes surgical resection with the goal of limited airway resection by means of lobectomy or pneumonectomy for larger or proximal lesions; endoscopic intrabronchial resection and yttrium aluminium garnet laser resection.^2^, ^5^ Contraindications to minimally invasive surgical approach include involvement of a spinal artery or medullar tumour involvement.^3^ Given these tumours are generally loosely attached with associated nerves, resection can be completed without significant damage. Although recurrence is rare with only <1%–2%, further studies are warranted to better understand schwannomas.^4^

## AUTHOR CONTRIBUTIONS

Primary authors; Syed Haq, Claire Friederick, and David Eapen. Data gathering and research assistance; Sidra Shah, Hoshimjon Begmatov, and FNU Sandesh. Editors; Syed H. Haq and Sreenivasa Chanamolu.

## CONFLICT OF INTEREST STATEMENT

None declared.

## ETHICS STATEMENT

The authors declare that appropriate written informed consent was obtained for the publication of this manuscript and accompanying images.

## Data Availability

Data sharing not applicable to this article as no datasets were generated or analysed during the current study.
